# Cross-cultural adaptation and validation for central sensitization inventory: based on Chinese patients undergoing total knee arthroplasty for knee osteoarthritis

**DOI:** 10.1186/s13018-023-04375-3

**Published:** 2023-12-13

**Authors:** Chao Xu, Shuxin Yao, Wei Wei, Haiyue Zhang, Jianbing Ma, Lei Shang

**Affiliations:** 1 Department of Health Statistics, Faculty of Preventive Medicine, Fourth Military Medical University, No.169. Changle West Rd, Xi’an, Shaanxi China; 2 Department of Knee Joint Surgery, Honghui Hospital, Xi’an Jiaotong University, No. 555 Youyi East Rd, Xi’an, Shaanxi China; 3 Department of Orthopedics, 989th Hospital of PLA, No. 2 Huaxia West Rd, Luoyang, Henan China

**Keywords:** Central sensitization inventory, Central sensitization, Knee osteoarthritis, Psychometrics, Total knee arthroplasty, Chinese

## Abstract

**Background:**

This study was conducted to develop a simplified Chinese version of the central sensitization inventory (CSI-CV) and to evaluate its reliability and validity.

**Methods:**

The CSI-CV was developed through a process involving the translation and back translation of the original CSI. Subsequently, experts reviewed and revised the content of the items to ensure their appropriateness. A total of 325 patients with knee osteoarthritis (KOA), who were scheduled to undergo total knee arthroplasty (TKA), completed the CSI-CV at a prominent orthopedic center in Xi'an, China. Afterward, a random selection of 100 participants was chosen for retesting after one week. The reliability and validity of the inventory were evaluated through exploratory factor analysis, correlation coefficient calculation and other methods.

**Results:**

The CSI-CV consists of 25 items in five dimensions (emotional distress, headache and jaw symptoms, physical symptoms, urological symptoms, and fatigue and sleep problems). The cumulative variance contribution rate was 75.3%, the Cronbach's α coefficient was 0.83, the Guttman split-half reliability coefficient was 0.88 and the intraclass correlation coefficient was 0.965. The CSI-CV scores correlated moderately with the total scores of the brief pain inventory (r = 0.506), Western Ontario and McMaster Universities Osteoarthritis Index (r = 0.466) and EuroQoL Group's five-dimension questionnaire (r = 0.576).

**Conclusions:**

The findings demonstrate that the CSI was successfully trans-culturally adapted into a simplified Chinese version (CSI-CV) that was reliable and valid for Chinese-speaking patients who awaiting TKA for KOA.

**Supplementary Information:**

The online version contains supplementary material available at 10.1186/s13018-023-04375-3.

## Background

Knee osteoarthritis (KOA) is one of the most common degenerative diseases in elderly populations, resulting in chronic persistent pain and functional disability [1]. The average crude prevalence of KOA among individuals aged over 55 years was 13.2% between the years 2008 and 2017 in China [2]. Despite the recognized effectiveness of total knee arthroplasty (TKA) as a surgical intervention for end-stage KOA, a notable percentage (approximately 10–20%) of patients still report dissatisfaction [3]. Persistent unexplained pain following TKA has emerged as one of the strongest factors associated with this dissatisfaction [4].

Over the past few decades, the concept of central sensitization (CS) has been developed. It is defined as an enhanced responsiveness of nociceptive neurons in the central nervous system to their normal or subthreshold afferent input [5]. CS has been recognized as a significant risk factor for persistent pain and patient dissatisfaction after TKA [6–12]. Therefore, assessing the patient's CS status before surgery is essential to enable early intervention if necessary [13].

Several attempts have been made to objectively quantify CS, such as Quantitative Sensory Testing (QST) [14, 15] and (f)MRI [16, 17]. While these tools can provide valuable information, they are often complex, time-consuming, and expensive [18]. To address these limitations, researchers are investigating the feasibility of utilizing more accessible and cost-effective methods, such as the central sensitization inventory (CSI). CSI is a self-report questionnaire, which identifies key symptoms associated with CS and quantifies the severity of these symptoms [19]. The questionnaire consists of two parts, Part A assesses 25 CS-related symptoms regarding the patient’s current conditions, while Part B investigates whether the patient has or had 10 central sensitivity syndromes (CSS) (fibromyalgia, chronic fatigue syndrome, temporomandibular joint disorder, irritable bowel syndrome, migraine or tension headaches, multiple chemical sensitivities, and restless leg syndrome, neck injuries, anxiety or panic attacks, depression) [19].

The CSI has been translated and validated in multiple countries for patients with chronic pain resulting from various causes [19–32]. Previously, Feng et al. translated and validated the traditional Chinese version of the CSI in Hong Kong [23]. Traditional Chinese is written and spoken in regions like Hong Kong, Macau, Taiwan, and Singapore. Simplified Chinese is the predominant official language in mainland China. This linguistic diversity reflects the cultural and geographical variations [33]. The majority of individuals in mainland China may encounter difficulties when it comes to reading and writing in traditional Chinese [33, 34]. Consequently, there is a pressing need to translate and culturally adapt the simplified Chinese version of the CSI. As such, the objective of this study is to translate, cross-culturally adapt, and validate the psychometric properties of the CSI in mainland Chinese KOA patients scheduled to undergo TKA. The aim is to establish a robust foundation for the application of the CSI in preoperative screening specifically for TKA procedures.

## Materials and methods

The medical ethics committee of Honghui Hospital approved this prospective observational study (No. 202105010). All experiments were performed in accordance with the Declaration of Helsinki. All participants provided written informed consent.

### Translation and cross-cultural adaptation

To culturally adapt the CSI, a translation and back-translation approach was employed [35]. Initially, the CSI was translated from English to simplified Chinese by two native Chinese speakers: an orthopedic surgeon proficient in English and a professional standard translator. The translation preserved the original English version's items and scoring instructions without any modifications. An experienced cross-cultural adaptation expert collaborated with the two translators to merge their translations into a unified version. Following that, two other English-speaking individuals with no medical background independently retranslated the preliminary unified version back into English. Back translation is an effectiveness verification process aimed at ensuring that the translated version accurately conveys the same item content as the original version. This step frequently highlights any ambiguities or unclear phrasing in the translation [36]. To further refine the simplified Chinese version of the CSI, a pretest was conducted with 50 elderly patients suffering from end-stage KOA. Their feedback was collected and considered during the reconciliation process, comparing the back-translated version with the original version. Subsequently, all researchers involved in the study deliberated upon the testing issues and developed the final version of the simplified Chinese CSI (CSI-CV).

### Participants

Between July 2021 and December 2022, patients with end-stage KOA who were scheduled to undergo TKA were recruited from the Department of Knee Joint Surgery at Honghui Hospital in Xi'an City, Shaanxi Province, China. The inclusion criteria for this study were as follows: (a) patients older than 18 years old with end-stage KOA who were scheduled to undergo primary unilateral TKA; (b) whose cognitive level meets the requirements for completing the questionnaire; (c) who were fluent in Mandarin at a conversational level; and (d) who consented to participate. Exclusion criteria were: (a) patients with a history of other vascular, nerve, muscle, and bone diseases that affect movement or produce pain symptoms, such as hemiplegia, fractures, ligament injuries, lower extremity vascular injuries, etc.; (b) Patients who have previously undergone TKA; (c) patients with serious diseases that affect daily life, such as coronary heart disease, asthma, mental illness, etc.

The self-assessment questionnaires were administered by a trained interviewer who instructed the participants to independently complete the questionnaire before TKA. After receiving the completed questionnaires, a thorough review was conducted to identify any missing items. In instances where items were found to be missing, participants were kindly requested to provide the necessary answers [37]. To ensure consistency, standardized instructions were given to participants regarding how to complete the missing items [22]. To evaluate test–retest reliability, 100 patients from the first interview were randomly selected and were asked to complete the CSI-CV again within an interval of 1 weeks, before TKA.

### Questionaries

All patients were required to complete the questionnaires as briefly described below.

#### Demographic information

Every participant was required to fill out the general demographic information questionnaire, which included age, body mass index, gender, employment status, living situation and educational level.

#### The CSI-CV

The CSI-CV consists of two sections: Part A and Part B [19]. Part A evaluates 25 symptom items. Participants rate the frequency of each symptom using a Likert scale ranging from 0 (never) to 4 (always), resulting in a total possible score of 100. Higher scores indicate a greater self-reported symptom burden. To aid clinical interpretation, five severity levels have been recommended: subclinical (0–29), mild (30–39), moderate (40–49), severe (50–59), and extreme (60–100) [38].

Part B is not scored and focuses on assessing 10 CSS-related diagnoses. Participants are asked two questions: (1) whether they have been previously diagnosed with each of these disorders by a doctor, and (2) the year of diagnosis.

#### The EuroQol five-dimensional questionnaire (EQ-5D)

The EQ-5D is a self-administered measurement instrument used to assess health-related quality of life. It comprises five domains (mobility, self-care, functional activity, pain/discomfort, and anxiety/depression) and is measured on a five-grade scale: no difficulties, slight difficulties, moderate difficulties, severe difficulties, and extremely severe difficulties [39, 40].

#### The brief pain inventory (BPI)

The BPI consists of two main scores: the pain severity score and the pain interference score [41]. The pain severity score is determined by four items that measure the patient's "most severe", "least severe" and "average" pain experienced in the past 24 h, as well as their current pain at the time of completing the questionnaire. Each item is rated on a scale of 0 to 10, ranging from "no pain" to "the most severe pain imaginable".

The pain interference score is calculated based on seven items that evaluate the extent to which pain affects various aspects of life. Each item is rated on a scale of 0 to 10, ranging from "no interference" to "complete interference".

#### The Western Ontario and McMaster Universities Osteoarthritis Index (WOMAC)

The WOMAC is divided into three domains: stiffness (two items), pain (five items), and physical function (17 items) [42]. Each item is rated on a scale of 0 to 10. The scores from the three domains are then combined to generate a total score, with higher scores indicating increased pain, stiffness, and impaired function.

### Sample size determination

To determine the appropriate sample size, we followed the recommendations for conducting factor analysis. These recommendations suggest having 4 to 10 participants per item [43] and a total sample size of more than 100 participants [44]. Therefore, the final sample size of this study was determined to be ≥ 250 cases.

### Statistical analysis

Descriptive statistics were used to summarize the demographic and clinical characteristics of the participants, with the mean and SD calculated for continuous variables, and percentages calculated for categorical variables. The Kolmogorov–Smirnov test was used to assess the normality of the CSI-CV, EQ-5D, WOMAC, and BPI total scores. T-tests and one-way analysis of variance (ANOVA) with Bonferroni correction were employed to compare CSI-CV scores across subjects with different characteristics. This analysis aimed to assess the discriminant validity of the scale. All statistical analyses were conducted using SPSS 24.0 (Chicago, IL). The significance level was set at 0.05.

#### Structural validity

Since the factorial structure of the CSI has been shown to vary in different language versions [20–30], structural validity of the CSI-CV was investigated with an exploratory approach to assess the number of factors. The adequacy for these analyses was calculated by the Kaiser–Meyer–Olkin test and Bartlett’s test of sphericity. An exploratory factor analysis (EFA) was conducted with the maximum likelihood method using a promax rotation. Factors were considered for eigenvalues > 1.0. The cut-off for the loadings should be set at 0.4 [45].

#### Criterion validity

The Pearson correlation coefficient was used to test the criterion validity of the CSI-CV with the EQ-5D, WOMAC and BPI. The correlation levels were set as follows: > 0.81, very strong correlation; 0.61–0.80, strong correlation; 0.41–0.60, moderate correlation; 0.21–0.40, weak correlation; and < 0.20, none or very weak correlation [46]. Before this analysis, based on assessment of the content of the items on the scales, we hypothesized that the CSI-CV scores correlated moderately with the total scores of the EQ-5D, WOMAC and BPI.

#### Internal consistency and test–retest reliability

Internal consistency indicates how closely related a set of items is as a group, which is usually measured by Cronbach's α coefficients. The coefficient α was set as follows: < 0.70, poor or unacceptable; 0.70–0.79, fair; 0.80–0.89, good; and ≥ 0.90, excellent [47]. Test–retest reliability was assessed using the intraclass correlation coefficient (ICC), which was derived from a 2-way analysis of variance in a random effect model. ICC values between 0.5 and 0.75 indicated moderate reliability, values between 0.75 and 0.9 indicated good reliability, and values greater than 0.9 suggested excellent reliability [48]. Guttman's split-half reliability coefficients were calculated to evaluate scale reliability and equivalence [37]. It is recommended that the interval between repeated measurements should be 1–2 weeks [49]. We decided to send the questionnaire again after an interval of 7 days.

#### Item screening

Items were screened based on their factor loadings of item scores and Cronbach's α coefficient. Item deletion was contemplated if the item’s factor loading was less than 0.4, and the Cronbach's α coefficient of the entire scale increased after removing the item compared to before its removal [37].

#### Measurement error

Standard error of measurement (SEM) is an indicator of absolute reliability [49]. The most common calculation method for this statistic is the following equation: SEM = SD·$\sqrt{(1-R)}$, SD = the sample standard deviation, and R = the calculated ICC [50].

#### Smallest detectable change

The smallest detectable change (SDC) reflects the smallest within-person change in score, calculated using the formula SDC(_95% CI_) = 1.96·$\sqrt{2}$·SEM [49].

#### Feasibility

Each participant was queried about any challenges encountered while completing the questionnaire. The feasibility was assessed by considering the percentage of participants who did not respond to certain items, as well as the overall time taken to complete the questionnaire.

## Results

### Participants

A total of 325 patients were included in the study. The demographic data of all the participants are shown in Table 1.

**Table 1:** Demographic data of participants

Items	Participants (*n* = 325)
Age (SD, range)	65.6 years (6.5, 45.0 to 79.0)
Body mass index (SD, range)	26.1 kg/m^2^ (2.9, 19.0 to 32.0)
*Gender*	
Male	96 (29.5%)
Female	229 (70.5%)
*Employment status*	
In work	210 (64.6%)
Be unemployed	20 (6.2%)
Retired	95 (29.2%)
*Living situation*	
Living alone	80 (24.6%)
With spouse	220 (67.7%)
With children	25 (7.7%)
*Education*	
Primary school or below	138 (42.5%)
Middle school	122 (37.5%)
High school or above	65 (20.0%)

*SD* standard deviation

### Cross-cultural adaptation and Item screening results

The forward and backward translations of the CSI into simplified Chinese were successfully completed, as described in Additional file 1, without encountering any major issues. All item’s factor loadings were above 0.4 (Table 3). Additionally, the Cronbach's α coefficient for the scale did not increase after removing any item. Therefore, no item needed to be removed following the screening process.

### Prevalence

The CSI scores among the 325 patients ranged from 8 to 62, with a mean of 29.6 (SD = 10.1). Of the 325 patients, 168 patients indicated subclinical, 119 patients indicated mild, and 38 patients indicated moderate or higher severity. As shown in Table 2, of the total 325 patients, 35 (10.8%) patients were diagnosed with CS-related diseases. Patients diagnosed with only one CSS (25 patients, 47.2 ± 6.9) or 2 CSSs (10 patients, 54.4 ± 4.9) scored higher on the CSI than those with no CSS diagnosis (290 patients, 27.2 ± 7.5; *P* < 0.01).

**Table 2:** Prevalence rates of CS severity levels and frequency of diagnoses

CSI-CV score	N (%)
Subclinical (0–29)	168 (51.7)
Mild (30–39)	119 (36.6)
Moderate (40–49)	18 (5.5)
Severe (50–59)	17 (5.2)
Extreme (> 60)	3 (0.9)
*Diagnoses*	
Restless leg syndrome	1 (0.3)
Chronic fatigue syndrome	2 (0.6)
Fibromyalgia	6 (1.8)
Temporomandibular joint disorder	0 (0)
Migraine or tension headaches	5 (1.5)
Irritable bowel syndrome	4 (1.2)
Multiple chemical sensitivities	1 (0.3)
Neck injury (including whiplash)	4 (1.2)
Anxiety or panic attacks	4 (1.2)
Depression	18 (5.5)

*CS* central sensitization, *CSI-CV* simplified Chinese version of the Central Sensitization Inventory

### Structural validity

**Fig. 1:**
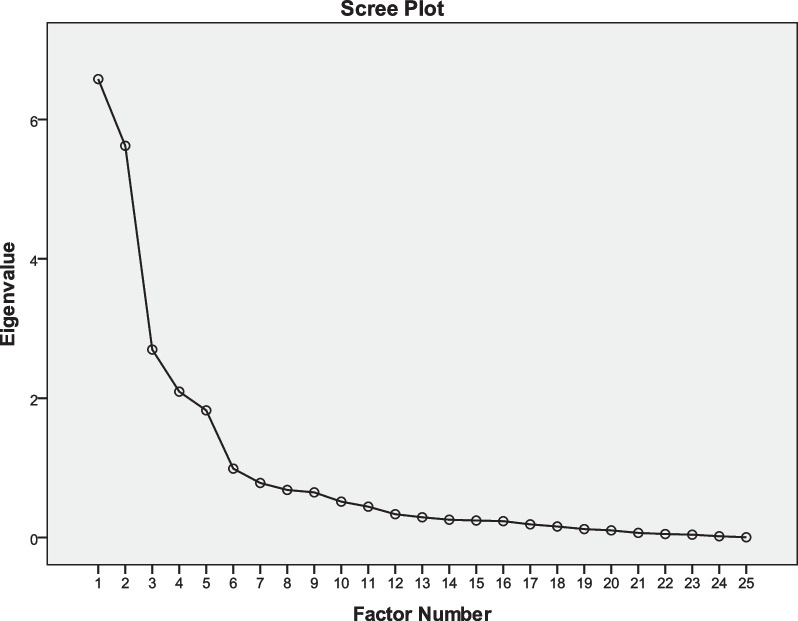
Scree plot of exploratory factor analysis

The results of the Kaiser–Meyer–Olkin test (0.784) and the Bartlett’s test of sphericity (*P* < 0.001) confirmed that the data was suitable for factor analysis. EFA and screen plot (Fig. 1) resulted in a five-factor model. The eigenvalues of the Factor 1 to Factor 5 were 6.58, 5.62, 2.70, 2.10 and 1.83, and the variance contribution rates of the Factor 1 to Factor 5 were 26.32%, 22.49%, 10.80%, 8.38% and 7.30%, respectively. The cumulative variance contribution rate was 75.3%. The factors matrix is presented in Table 3. Items 3, 5, 6, 13, 15, 16, 23 and 24 were loaded on factor 1, which was named “Emotional Distress”. Factor 2 consisted of items 4, 7, 10 and 19, and was named “Headache and Jaw Symptoms”. Factor 3 consisted of items 2, 9, 14, 17, 18 and 20, and was named “Physical Symptoms”. Factor 4 consisted of items 11, 21 and 25, and was named “Urological Symptoms”. Factor 5, consisting of items 1, 8, 12, and 22, exhibited a significant association with fatigue and sleep-related difficulties. Consequently, we appropriately labeled this factor as "Fatigue and Sleep Problems".

**Table 3:** Factor loadings of the EFA with promax rotation

Item no.	Question	F1	F2	F3	F4	F5	Original version [19]	Hongkong version [23]
5	Diarrhea/constipation	0.939					F3	Not loading
16	Sad or depressed	0.938					F1	F1
6	Need help with daily activity	0.935					F3	Not loading
15	Stress makes symptoms worse	0.930					F1	Not loading
13	Difficulty concentrating	0.929					F1	F1
3	Anxiety attacks	0.908					F1	F1
24	Trauma as a child	0.768					F1	Bladder and teeth grinding disorders
23	Poor memory	0.697					F1	Concentration and memory problem
19	Pain in jaw		0.955				F2	Not loading
7	Sensitive to bright lights		0.927				F2	Hypersensitivity syndrome
4	Grind/clench teeth		0.922				F2	Bladder & teeth grinding disorders
10	Headaches		0.896				F2	Not loading
17	Low energy			0.854			F3	Concentration and memory problem
2	Muscles stiff/achy			0.769			F3	F3
14	Skin problems			0.709			F3	Not loading
20	Certain smells make dizzy			0.676			F2	Not loading
9	Pain all over body			0.591			F3	F3
18	Tension neck and shoulder			0.512			F3	F3
11	Bladder/urination pain				0.907		F4	Bladder & teeth grinding disorders
25	Pelvic pain				0.896		F4	Not loading
21	Urinate frequently				0.822		F4	Hypersensitivity syndrome
22	Restless legs					0.857	F3	Not loading
12	Do not sleep well					0.807	F3	Not loading
8	Easily tired with physical activity					0.741	F3	F3
1	Unrefreshed in morning					0.565	F3	Not loading

*EFA* exploratory factor analysis, *F1* Emotional Distress, *F2* Headache and Jaw Symptoms, *F3* Physical Symptoms, *F4* Urological Symptoms, *F5* Fatigue and Sleep Problems

### Criterion validity

The correlations between the CSI-CV and EQ-5D, BPI and WOMAC were showed in Table 4. As hypothesized, the results revealed positive correlations between CSI-CV scores and the scores of EQ-5D, BPI, and WOMAC.

**Table 4:** Results of bivariate correlations between CSI-CV and related scales

Variance	Mean (SD)	Correlation coefficient	*P*-value
Health-related QOL (EQ-5D)	13.1 (3.6)	0.576	< 0.001
BPI	43.4 (17.0)	0.506	< 0.001
WOMAC	91.5 (39.1)	0.466	< 0.001

*SD* standard deviation

### Internal consistency and test–retest reliability

The CSI-CV exhibited a strong level of internal consistency (Cronbach’s α = 0.83), with individual factor scores ranging from 0.76 to 0.98. Furthermore, the Guttman split-half reliability coefficient was determined to be 0.88, with individual factor scores ranging from 0.59 to 0.92. These findings indicate that each item within the measure showed significant correlation with the overall factor being assessed. Test–retest reliability was excellent with an ICC of 0.965. All factors of the CSI had an excellent ICC greater than 0.90. The SEM was 1.89 points (Table 5).

**Table 5:** Cronbach’s α, intraclass correlation coefficients, and Guttman split-half coefficient of test–retest reliability

Item no.	Questions	Cronbach’s α	ICC	Guttman split-half coefficient
Sum	Total score	0.83	0.965	0.88
Factor 1	Emotional Distress	0.96	0.965	0.92
Factor 2	Headache and Jaw Symptoms	0.98	0.948	0.98
Factor 3	Physical Symptoms	0.79	0.910	0.79
Factor 4	Urological Symptoms	0.86	0.971	0.78
Factor 5	Fatigue and Sleep Problems	0.76	0.966	0.71

*ICC* intraclass correlation coefficients

### SDC

The SDC, representing the smallest change of value for a scale that could be considered an actual change rather than measurement of error, was 5.22 points.

### Feasibility

All participants completed Part A of the CSI-CV without any difficulties, and there were no issues with missing or multiple answers. However, it is worth noting that some patients experienced difficulties in completing Part B. These challenges were attributed to their limited familiarity with certain diagnoses. The average time for completing the questionnaires was 6.4 ± 1.3 min.

### Discriminant validity

There was no significant difference in CSI scores between two groups of patients of different ages. The total scores of the CSI-CV differed significantly according to gender, employment status, living situation and education. The results indicated that women scored significantly higher than men on the CSI-CV scale. Additionally, unemployed individuals obtained significantly higher scores compared to working individuals and retirees. Furthermore, individuals living alone achieved significantly higher scores than those living with their spouse or children. Lastly, individuals with lower education levels obtained significantly higher scores compared to those with higher education levels (Table 6).

**Table 6:** CSI-CV scores in different populations

Factor		N	Total score of CSI	*P* value
Age	≥ 70 years old	121	30.0 ± 9.4	0.545
	< 70 years old	204	30.5 ± 10.5	
Gender	Male	96	27.5 ± 8.1^a^	0.018
	Famale	229	30.4 ± 10.8^a^	
Employment status	In work	210	30.2 ± 9.1^a^	< 0.001
	Be unemployed	20	43.4 ± 10.7^a^	
	Retired	95	25.3 ± 9.3^a^	
Living situation	Living alone	80	31.6 ± 11.1^a^	0.035
	With spouse	220	29.2 ± 9.9	
	With children	25	26.0 ± 7.3^a^	
Education	Primary school or below	138	31.9 ± 10.1^a^	0.001
	Middle school	122	27.5 ± 10.3^a^	
	High school or above	65	28.4 ± 9.0	

Values are expressed as means ± standard deviation. ^a^ Items with significant differences. *P* value represents the comparison result of T-tests or ANOVA with Bonferroni correction

## Discussion

The objective of this study was to develop a culturally adapted simplified Chinese version of the CSI and assess its psychometric properties among patients undergoing TKA for KOA. Among these patients, there is considerable variation in the presence and intensity of CS. Previous research has shown that CS predicts poor treatment outcomes in TKA patients [6–10]. This emphasizes the critical importance of individualized assessments to precisely evaluate each patient's condition [51]. Such screening can provide valuable information that enables early intervention [13] before or intensive treatment after TKA. Furthermore, by identifying CS in KOA patients before surgery, surgeons can engage in shared decision-making with patients, discussing the potential implications of CS on their postoperative recovery and setting realistic expectations [5, 52, 53].

In our study, EFA revealed a 5-factor model for the CSI-CV, which aligns with the findings from the Japanese [20] and French [31] versions. However, the English version demonstrated a 4-factor model [19], the Spanish version showed a 1-factor model [27], and the Korean version resulted in a 6-factor model [30].

Table 3 displays the factor loading of each item across different versions of the CSI. The observed variations in the factor structure between this study and previous studies conducted with other language versions [19, 27, 30] underscore the influence of cultural and linguistic factors on the conceptualization of CS in different populations [20, 30]. These findings emphasize the importance of adapting assessment tools to specific cultural settings to ensure their validity and reliability in measuring the construct of interest [20].

The ICC indicated that the CSI-CV has excellent reliability, at 0.965. The results corroborated earlier reports on English (0.817) [19], Japanese (0.85) [20], Korean (0.941) [30], French (0.91) [31] and Brazilian (0.91) [32] versions. In addition, the CSI-CV demonstrated good criterion validity when compared to the BPI, the WOMAC, and the EQ-5D. The level of correlation observed was similar to that of the Korean and Japanese versions of the CSI [20, 30]. All of these findings collectively support the strong psychometric properties of the CSI-CV.

The CSI-CV exhibited a high level of internal consistency with a Cronbach's alpha of 0.83. Additionally, the 25 sub-items displayed high Cronbach's α values ranging from 0.82 to 0.83. These values were consistent with the English (0.87) [19], Japanese (0.89) [20], Korean (0.94) [30], Spanish (0.87) [27], and Italian (0.87) [29] versions. This indicates that the CSI remains stable internal consistency across different cultural contexts.

Only a few of the initial validation studies [22, 25, 26] computed the SEM and the SDC. The SEM values ranged from 0.31 [25] to 4.14 [22], while the SDC values ranged from 0.86 [25] to 11.48 [22]. In this study, the values for SEM and SDC are 1.89 and 5.22, respectively, falling between the two ranges mentioned. The differences in these values could be attributed to specific factors. In the Nepali study [25], participants were provided with baseline measurement values during the follow-up, and the CSI was administered face-to-face. These factors may have influenced participant responses and introduced potential biases. Conversely, the longer interval between measurements in the German study [22] could have minimized memory effects, potentially increasing the values of SEM and SDC. Based on the current findings, any change score greater than 5.2 units (out of 100) may be considered a true change for the CSI-CV.

In addition to its psychometric properties, the feasibility of the CSI-CV was also evaluated in this study. The time required to complete the CSI-CV was found to be relatively short, with participants spending an average of 6.4 ± 1.3 min to complete the questionnaire. The brevity of the CSI-CV allows for efficient administration, reducing participant burden and increasing the likelihood of compliance.

The CSI scores in the present study ranged from 8 to 62, with a mean of 29.6 ± 10.1. These scores were lower than those reported in Germany (43.6 ± 15.0) [22], Korea (33.4 ± 15.7) [30], Serbia (38.3 ± 15.7) [26], Italy (35.3 ± 14.6) [29], Brazil (45.4 ± 17.4) [32], and Hong Kong of China (36.4 ± 13.1) [23]. On the other hand, the scores in this study were higher compared to the Japan [20] and Nepal [25] studies. In addition, in the present study, the proportion of CSS diagnoses was found to be 10.8%, which is lower than the proportions reported in most studies conducted in other areas ranging from 13 [23] to 56% [29].

These variations in CSI scores and the prevalence of CSS diagnoses can be attributed to various factors, such as cultural, racial, and medical differences among the patient populations [51]. Cultural and societal attitudes towards mental health can influence patients' willingness to acknowledge and disclose their own mental sensitivities. In this study, the patients were recruited from underdeveloped inland areas in northwest China, where a relatively conservative social atmosphere may discourage individuals from openly admitting their mental health issues. Additionally, limited access to advanced medical resources has led to underdiagnosis of CSS in many patients. These disparities underscore the importance of exercising caution when interpreting scale scores in diverse cultural contexts, and suggest that the clinical threshold for identifying CS may need to be adjusted to accommodate the unique social environment in China [23].

Furthermore, it is important to note that the present study focused specifically on patients undergoing TKA due to KOA. This distinguishes it from previous studies that included a broader range of conditions such as musculoskeletal pain, fibromyalgia, and acute injury pain [20–30]. The specific patient population targeted in this study may have contributed to the observed differences in CSI-CV scores.

The CSI total score exhibits a substantial disparity among individuals with different demographic characteristics. Specifically, women tend to have significantly higher CSI scores compared to men, aligning with previous research findings indicating that women with acute pain are more likely to experience persisting pain [51, 54]. Furthermore, unemployed individuals, those living alone, and those with lower education levels also display higher CSI scores, likely due to the increased economic and social pressures they face. Consequently, it is imperative that healthcare professionals prioritize these specific groups and offer proactive measures, such as early interventions like Duloxetine [13, 55, 56] or Pregabalin [57–59] during the perioperative period.

Numerous limitations of the present study should be acknowledged. Firstly, all participants were recruited from a single hospital, predominantly representing patients in northwestern China. Given the vast land area of China, the generalizability of the findings to other regions of mainland China may be limited. Secondly, the assessment of CSS was based on self-report questionnaires, which introduces the potential for response bias. Thirdly, we did not directly measure CS using QST in our study. Further research is required to investigate the validation of the CSI-CV, which could involve incorporating QST as a complementary measure. Lastly, the study did not include healthy individuals, warranting future research to investigate whether the CSI-CV can effectively differentiate individuals with and without CS.

## Conclusions

In conclusion, the linguistic translation and cultural adaptation of the CSI from the original English version to simplified Chinese were successfully completed, ensuring the equivalence of all items. The CSI-CV demonstrated strong psychometric properties, including reliability, validity, and ease of understanding. These findings provide robust evidence supporting the use of the CSI-CV as a reliable instrument for screening CS in Chinese-speaking patients with KOA prior to undergoing TKA.

### Supplementary Information

**Additional file 1**. Simplified Chinese version of the Central Sensitization Inventory.

## Data Availability

The datasets used and/or analyzed during the current study are available from the corresponding author on reasonable request.

## Abbreviations

CSI-CV: Simplified Chinese version of the central sensitization inventory
KOA: Knee osteoarthritis
TKA: Total knee arthroplasty
EFA: Exploratory factor analysis
CS: Central sensitization
QST: Quantitative sensory testing
CSI: Central sensitization inventory
CSS: Central sensitivity syndromes
EQ-5D: The EuroQol Five-Dimensional Questionnaire
BPI: Brief Pain Inventory
WOMAC: The Western Ontario and McMaster Universities Osteoarthritis Index
ANOVA: One-way analysis of variance
ICC: Intraclass correlation coefficient
SEM: Standard error of measurement
SDC: Smallest detectable change
SD: Standard deviation
