# Supplementary material for: Cross-cultural adaptation and validation for central sensitization inventory: based on Chinese patients undergoing total knee arthroplasty for knee osteoarthritis
Source: J Orthop Surg Res. 2023 Dec 13;18:960. doi: 10.1186/s13018-023-04375-3 (PMC10717624; doi:10.1186/s13018-023-04375-3)
Supplement: Supplementary file 1 — Additional file 1. Simplified Chinese version of the Central Sensitization Inventory. [file 13018_2023_4375_MOESM1_ESM.docx]

**simplified Chinese version of the Central Sensitization Inventory**

**(Part A:** 25 symptom items**)**

**请阅读下列描述，在后面的选项中圈出最符合您实际情况的一个答案。**

|  |  | 0 | 1 | 2 | 3 | 4 |
| --- | --- | --- | --- | --- | --- | --- |
| 1 | 睡醒后感觉很疲惫、乏力。 | 从不 | 很少 | 有时 | 经常 | 总是 |
| 2 | 浑身的肌肉感到僵硬、酸痛。 | 从不 | 很少 | 有时 | 经常 | 总是 |
| 3 | 感到焦虑、恐慌。 | 从不 | 很少 | 有时 | 经常 | 总是 |
| 4 | 睡觉时磨牙。 | 从不 | 很少 | 有时 | 经常 | 总是 |
| 5 | 有腹泻和/或便秘的问题。 | 从不 | 很少 | 有时 | 经常 | 总是 |
| 6 | 进行日常活动需要别人帮助。 | 从不 | 很少 | 有时 | 经常 | 总是 |
| 7 | 对亮光很敏感。 | 从不 | 很少 | 有时 | 经常 | 总是 |
| 8 | 体力劳动时很容易累。 | 从不 | 很少 | 有时 | 经常 | 总是 |
| 9 | 感觉浑身疼痛。 | 从不 | 很少 | 有时 | 经常 | 总是 |
| 10 | 有头痛。 | 从不 | 很少 | 有时 | 经常 | 总是 |
| 11 | 感觉膀胱不适和/或排尿时灼痛。 | 从不 | 很少 | 有时 | 经常 | 总是 |
| 12 | 睡眠质量不佳。 | 从不 | 很少 | 有时 | 经常 | 总是 |
| 13 | 很难集中精神。 | 从不 | 很少 | 有时 | 经常 | 总是 |
| 14 | 有皮肤问题，如干燥脱皮、瘙痒或起疹。 | 从不 | 很少 | 有时 | 经常 | 总是 |
| 15 | 感到压力时会加重躯体的不适感。 | 从不 | 很少 | 有时 | 经常 | 总是 |
| 16 | 感到悲伤、抑郁。 | 从不 | 很少 | 有时 | 经常 | 总是 |
| 17 | 感觉缺乏活力。 | 从不 | 很少 | 有时 | 经常 | 总是 |
| 18 | 感到颈肩部肌肉紧张。 | 从不 | 很少 | 有时 | 经常 | 总是 |
| 19 | 下巴处感到疼痛。 | 从不 | 很少 | 有时 | 经常 | 总是 |
| 20 | 闻到一些特殊的气味时，如香水，感到头晕、恶心。 | 从不 | 很少 | 有时 | 经常 | 总是 |
| 21 | 有尿频的问题。 | 从不 | 很少 | 有时 | 经常 | 总是 |
| 22 | 夜间入睡时，下肢感到难以名状的不适感，需要不停的活动。 | 从不 | 很少 | 有时 | 经常 | 总是 |
| 23 | 记忆力变差。 | 从不 | 很少 | 有时 | 经常 | 总是 |
| 24 | 小时候受过创伤。 | 从不 | 很少 | 有时 | 经常 | 总是 |
| 25 | 骨盆部位感到疼痛。 | 从不 | 很少 | 有时 | 经常 | 总是 |
|  | | | | | 总分= | |

**simplified Chinese version of the Central Sensitization Inventory**

**(Part B:** 10 central sensitivity syndromes related diagnoses**)**

**您是否被医生诊断为患有下列疾病？请对应做好标记，曾被诊断的话请注明诊断时间。**

|  |  | 否 | 是 | 诊断时间 |
| --- | --- | --- | --- | --- |
| 1 | 不宁腿综合征 |  |  |  |
| 2 | 慢性疲劳综合征 |  |  |  |
| 3 | 纤维肌痛 |  |  |  |
| 4 | 颞下颌关节紊乱综合征 |  |  |  |
| 5 | 偏头痛或紧张性头痛 |  |  |  |
| 6 | 肠易激综合征 |  |  |  |
| 7 | 化学物质敏感综合症 |  |  |  |
| 8 | 颈部损伤（包括扭伤） |  |  |  |
| 9 | 焦虑或恐慌发作 |  |  |  |
| 10 | 抑郁症 |  |  |  |
